# miR-1-Mediated Induction of Cardiogenesis in Mesenchymal Stem Cells via Downregulation of Hes-1

**DOI:** 10.1155/2013/216286

**Published:** 2012-12-20

**Authors:** Feng Huang, Liang Tang, Zhen-fei Fang, Xin-qun Hu, Jia-yi Pan, Sheng-hua Zhou

**Affiliations:** Department of Cardiology, The Second Xiangya Hospital of Central South University, Hunan, Changsha 410011, China

## Abstract

MicroRNAs (miRNAs, miRs) have the potential to control stem cells fate decisions. The cardiac- and skeletal-muscle-specific miRNA, miR-1, can regulate embryonic stem cells differentiation to cardiac lineage by suppressing gene expression of alternative lineages. Accordingly, we hypothesized that overexpression of miR-1 may also promote cardiac gene expression in mesenchymal stem cells. Since Notch signaling could inhibit muscle differentiation, a process in contrast with the effect of miR-1, miR-1-mediated repression of Notch signaling may contribute to the observed effects of miR-1 in mesenchymal stem cells. Thus, mesenchymal stem cells were infected by lentiviral vectors carrying miR-1, and cells expressing miR-1 were selected. Alterations in Notch signaling and cardiomyocyte markers, Nkx2.5, GATA-4, cTnT, and CX43, were identified by Western blot in the infected cells on days 1, 7, and 14. Our study showed that the downstream target molecule of Notch pathway, Hes-1, was obviously decreased in mesenchymal stem cells modified with miR-1, and overexpression of miR-1 promotes the specific cardiac gene expression in the infected cells. Knockdown of Hes-1 leads to the same effects on cell lineage decisions. Our results indicated that miR-1 promotes the differentiation of MSCs into cardiac lineage in part due to negative regulation of Hes-1.

## 1. Introduction 

Stem cell transplantation has been extensively investigated as a therapy to regenerate cardiac tissue after myocardial infarction. Mesenchymal stem cells (MSCs) can be easily isolated and expanded, and they possess neurogenic, chondrogenic, adipogenic, osteogenic, and myogenic properties under specific differentiating conditions [1, 2]. Furthermore, these cells have a stable genetic background and low risk of immune rejection. As a result, they are often used as seeding cells in tissue engineering and stem cell therapy. Precise regulation of cell fate decisions is a prerequisite for future therapeutic use of MSCs. Transcription factors that regulate pluripotency or lineage-specific gene and protein expression have been a major focus of stem cells research. Numerous signaling pathways, including Wnt, BMP, and Notch signaling pathways, regulate cell fate decisions during MSC differentiation and can be utilized to influence lineage choices *in vitro* [3–5]. Notch signaling plays an essential role in a variety of biological processes, including cell differentiation, cell fate specification, and patterning during embryonic and postnatal development [6]. In mammals, the Notch signaling pathway is comprised of five transmembrane ligands: delta-like- (Dll-) 1, (Dll-)3, (Dll-)4, Jagged (Jag-1), and Jagged (Jag-2), four transmembrane receptors: Notch-1, -2, -3, and -4, and downstream target genes, such as bHLH (basic helix-loop-helix) proteins: Hes (hairy/enhancer of split) and Hey (Hes-related protein). The pathway is crucial for cell-to-cell interaction during cardiovascular development and may influence cardiac differentiation, proliferation, and apoptotic events [7–9].

In addition to the numerous transcription factors and signaling molecules that control development of cardiac cells [10], microRNAs (miRNAs, miRs) also play critical roles in cardiac differentiation [11–13]. These small noncoding RNAs are naturally occurring molecules that are transcribed in the nucleus, often under the control of specific enhancers. They are processed by the RNAses Drosha/DGCR8 and Dicer into mature ~22 nucleotide RNAs, which can repress target mRNA expression by binding to miRNA regulatory elements (MREs) via Watson-Crick base pairing between the miRNA “seed region” and sequences commonly located in the 3′-untranslated region (3′-UTR) of target mRNAs [11, 14–16]. Previous studies have revealed that miRNAs play an important role in various cellular processes, including proliferation, differentiation, apoptosis, and development [17–19]. Some of these small RNAs are expressed in a lineage-specific fashion and, thus, have the potential to control stem cell fate decisions [20, 21]. Further studies have demonstrated that miRNAs contribute to the regulation of various signaling pathways via the repression of target genes, which results in the regulation and modulation of signal transduction [22]. MiR-1, a cardiac- and skeletal-muscle-specific miRNA, is involved in muscle differentiation and maintenance of muscle gene expression in both mammals and flies [11–13]. Notch signaling promotes neural differentiation and inhibits muscle differentiation in embryonic stem cells (ESCs) [23, 24], effects that are opposite to those of miR-1. Previous work by Kwon et al. showed that miR-1 directly represses the Notch ligand delta in *Drosophila* [12]. Three orthologs of *Drosophila* delta have been identified in mice: Dll-1, Dll-3, and Dll-4. Recently, Ivey and his colleagues demonstrated a new role for miRNAs in regulating ESCs differentiation. They found that miR-1 directs mesoderm formation from ESCs and regulates differentiation to cardiac lineage by suppressing gene expression of alternative lineages. This effect is partly due to miR-1 directly repressing the Notch ligand Dll-1 [25]. Accordingly, we hypothesized that overexpression of miR-1 may lead to similar effects in the culture MSCs.

## 2. Materials and Methods

### 2.1. Animal Ethics

All procedures were performed in accordance with guidelines of Laboratory Animal Care formulated by the National Society for Medical Research and conformed to the Guide for the Care and Use of Laboratory Animals published by the US National Institutes of Health (NIH Publication no. 85-23, revised 1996). 

### 2.2. Primary Culture and Characterization of MSCs

6-week-old male C57BL/6 mice were sacrificed by cervical dislocation. The hind legs and vertebrae were dissected and carefully separated from adherent tissues. After the tips of each bone were removed, bone marrow was collected by flushing out the content of femurs and tibias with phosphate buffered saline (PBS). The collected cells were cultured in complete medium, consisting of Dulbecco's Modified Eagle's Medium with Nutrient Mixture F12 (DMEM/F12; GIBCO), 10% fetal bovine serum (GIBCO), and 1% penicillin/streptomycin (GIBCO). The cultured cells were analyzed by fluorescence-activated cell sorting (FACS) as previously described [26]. The differentiation of MSCs *in vitro* towards the adipogenic and the osteogenic lineage is shown as previously described [27, 28].

### 2.3. Recombinant Lentiviral Vectors Construction, Cells Infection, and Stable Cell Line Generation

To produce recombinant lentiviral vectors encoding miR-1, mature miR-1, TRE promoter, and enhanced green fluorescent protein (eGFP) sequences were inserted into plasmids to produce pUp-TRE, pDown-miR-1, and pTail-IRES/eGFP; scramble sequence was set as negative control. pLV.EX3d.P/puro-TRE > miR-1 > IRES/eGFP was obtained with incubation of donors and accepter vectors catalyzed by LR Clonase (Gateway LR Clonase Plus Enzyme Mix, Invitrogen). Plasmid was then sequenced and purified for lentivirus envelope. Envelope helper plasmids: pLV/helper-SL3, pLV/helper–SL4, and pLV/helper–SL5, with pLV. EX3d.P/puro-TRE-miR-1-IRES/eGFP or pLVrtTA/neo which contains the imperative elements for virus packaging, were cotransfected into 293T cells with lipofectamine 2000, according to the manufacturer's instructions (Invitrogen) for generation of Lenti-miR-1-eGFP/puro or Lenti-rtTA/neo, respectively. To perform lentiviral infections, MSCs were firstly treated by Lenti-rtTA/neo; 48 h later, infected cell populations were selected in 0.5 mg/mL neomycin and refresh medium every two days. Selection was terminated when control cells were completely dead and antibiotic free medium was used for propagation. Neomycin resistant cells were infected by Lenti-miR-1-Puro/GFP and grown with 2 *μ*g/mL puromycin. Double resistance cells were then ultimately obtained, and 2 *μ*g/mL doxycycline was added to medium and intrigue expression of miR-1. MSCs infected with miR-1 recombinant lentiviral vectors carrying GFP were named MSCs^miR-1^, and MSCs infected with mock lentiviral vectors carrying GFP were named MSCs^null^, which used to determine whether the mock vectors without carrying miR-1 could influence the molecules of cells signaling pathway or cell lineage decisions compared to the control cells—MSCs.

To determine whether repression of Hes-1 could account for a subset of the effects of miR-1 on cell lineage decisions, we used the same way to construct lentiviral vectors carrying Hes-1 to transfect MSCs. To produce recombinant lentiviral vectors encoding Hes-1-shRNA, three types of plasmids: pAJ-U6-shRNA-CMV-Puro/GFP, psPAX2 (gag/pol element), and pMD2.G (VSVG element) were transfected into 293T cells according to the instructions. After 48 h transduction, infected MSCs were selected with 2 *μ*g/mL puromycin until mock-transfected cells died and were maintained under selection pressure throughout the generation of stable Hes-1-shRNA line. Cells transfected with Hes-1-shRNA recombinant lentiviral vectors were named MSCs^Hes-1-shRNA^. Stable shRNA line was cultured in a 5% CO_2_-humidified incubator at 37°C.

### 2.4. Quantitative Real-Time PCR (qRT-PCR) Identify the Efficiency of Gene Transfection

Total RNA was extracted from each sample with Trizol reagent (Invitrogen) according to the manufacturer's instructions. Expression of miR-1 was detected by qRT-PCR using All-in-One miRNA qRT-PCR Detection Kit (GeneCopoeia) and All-in-One miRNA qPCR Primer (GeneCopoeia). The primer sequence is 5′-CAGTCTGGCGAGAGAGTTCC-3′. The levels of miR-1 transcripts were normalized to the control U6 mRNA, which primer sequence was 5′-TCGTGAAGCGTTCCATATTTTTAA -3′. 

To test the expression of Hes-1 in the transfected cells, 2 *μ*g of total RNA extracted from each sample was reversed transcribed into first-strand cDNA by RevertAid First Strand cDNA Synthesis Kit (Fermentas), according to the manufacturer's instructions. The synthesized cDNA was used for real-time quantitative PCR analysis of Hes-1 mRNA expression with SYBR Premix Ex Taq (TaKaRa). The sense sequence of Hes-1 primers was 5^'^-AGAAGAGGCGAAGGGCAAGA-3′, whereas the antisense sequence was 5′-CGGAGGTGCTTCACAGTCAT-3′. Expression levels were quantified by normalizing the values relative to the mouse housekeeping gene: *β*-actin content, which primer sequence was 5′-CAGCCTTCCTTCTTGGGTAT-3′, and the antisense sequence was 5′-TGGCATAGAGGTCTTTACGG-3′. Relative gene expression was calculated by the 2^−ΔΔCT^ method [29]. 

### 2.5. Western Blot Analysis

Cells were washed twice with icecold PBS and were extracted with lysis buffer containing 50 mM Tris (pH 7.6), 150 mM NaCl, 1% TritonX-100, 1% deoxycholate, 0.1% SDS, 1 mM PMSF, and 0.2% Aprotinin (Sigma). The extract was centrifuged at 12,000 rpm for 15 min at 4°C to remove cellular debris. Protein concentrations were determined by the Bradford method (Bio-Rad Laboratories). Twenty micrograms of protein sample were heated to 95°C for 5 min, run in 10% SDS-PAGE gels, and transferred to PVDF membrane (Millipore) by using the semidry transfer method. The membranes were blocked for 1 h in Tris-buffered saline containing 0.01% Tween 20 with 10% nonfat dried milk and incubated overnight at 4°C with the relevant antibodies (Santa Cruz Biotechnology)—anti-Notch1, anti-Notch2, anti-Notch3, anti-Notch4, anti-Dll1, anti-Dll3, anti-Dll4, anti-Jag1, anti-Jag2, anti-Hes1, anti-Hes2, anti-Hey1, anti-Hey2, anticardiac troponin T (anti-cTnT), anti-NK2-transcription-factor-related locus 5 (anti-Nkx2.5), anti-GATA-4, and anti-connexin 43 (anti-CX43) antibody. After washing, the membranes were incubated with the secondary antibody, a peroxidase-conjugated anti-IgG (Bio-Rad Laboratories) for 1 h. All bands from Western blot were analyzed using Image J software (version 1.6 NIH) to verify the relative levels of Notch signaling and cardiomyocyte-specific markers compared to *β*-actin.

### 2.6. Statistical Analysis

Data were presented as mean values and standard deviation. A method of ANOVA (analysis of variance) with Scheffe's post hoc test was used to identify differences among all groups. *P* values of less than 0.05 were considered to be statistically significant.

## 3. Results 

### 3.1. Phenotypic Characterization and Differentiation Capacity of Cells

Cells were scattered in a number of colony distributions 3 days after planting. At days 8-9, the bottle was covered with long spindle-like cells. The passaged cells (mostly fibroblast-like cells) were uniformly distributed and covered the bottom every 4-5 days. The 3rd passage of cells highly expressed the MSCs surface marker molecules, CD29 and CD90, and negatively expressed the blood cell surface molecules, c-kit, CD34, and CD45. These cells were differentiated *in vitro* using adipogenic and osteogenic induction media. Following 3 weeks of adipogenic induction, the cells stained positive for Oil Red O, showing a lipid-laden adipocyte phenotype. Similarly, when induced with osteogenic induction medium for 3 weeks, these cells showed osteogenesis upon staining with Von Kossa for calcium deposits. These results demonstrated that the stem cells possess pluripotent of differentiation ability.

### 3.2. Efficiency of Gene Transfection and miR-1 Expression

**Figure 1:**
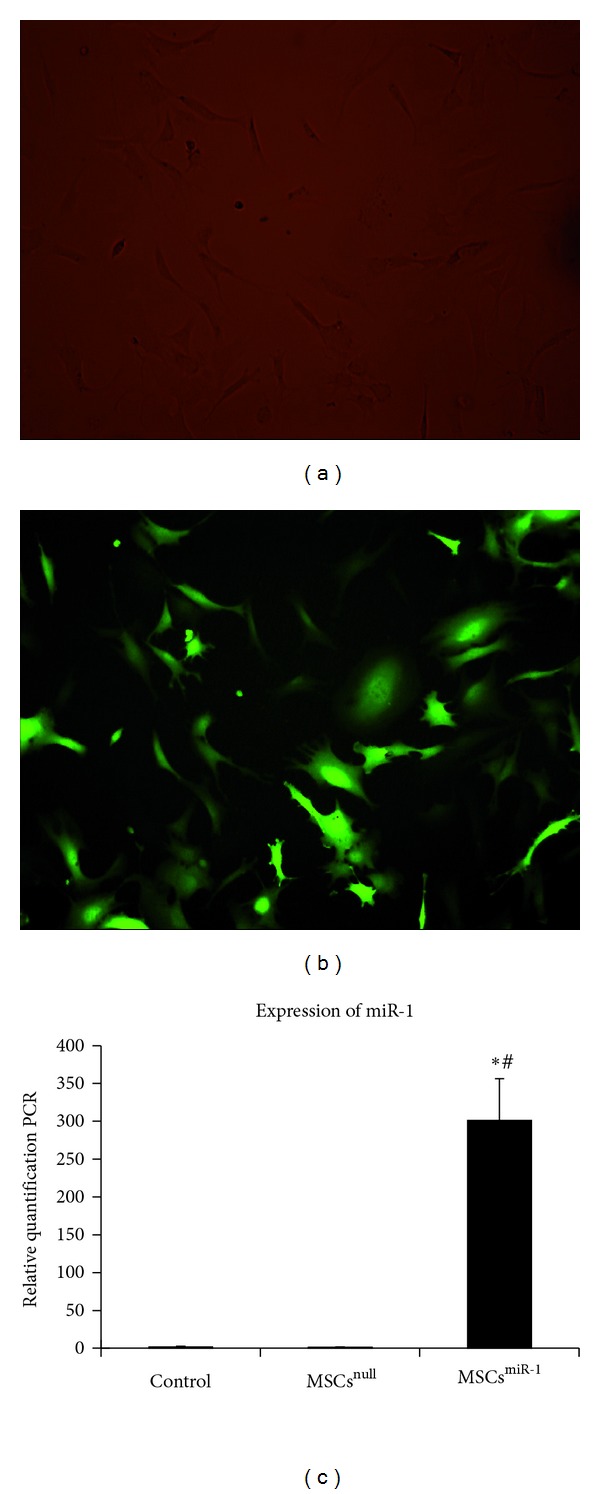
MSCs with fluorescence after transfection with lentiviral vectors and efficiency of miR-1 transfection. (a) Visible light field; (b)  green fluorescence field (Original magnification × 200 in (a) and (b)); (c) the efficiency of miR-1 transfection was analyzed by qRT-PCR, evident miR-1 expressed in MSCs^miR-1^, barely expressed in MSCs^null^ and control group (control = MSCs; MSCs^null^ = MSCs infected with mock lentiviral vectors without miR-1; MSCs^miR-1^ = MSCs infected with miR-1 recombinant lentiviral vectors; compared to control group, *P < 0.01; compared to MSCs^null^, ^#^ *P* < 0.01).

After infection with miR-1 recombinant lentiviral vectors, MSCs overexpressed GFP (Figures 1(a) and 1(b)). Quantitative real-time PCR test indicated that the efficiency of gene transfection in the MSCs^miR-1^ group was similar to that in the MSCs^null^ group (91.2% versus 90.3%). The expression of miR-1 in the MSCs^miR-1^ group was 212-fold higher than that in the MSCs group (*P* < 0.01) and 226-fold higher than expression in MSCs^null^ group (*P* < 0.01)  (Figure 1(c)). 

### 3.3. MiR-1 Downregulates Hes-1 and Promotes the Expression of Cardiomyocyte-Specific Makers in MSCs

**Figure 2:**
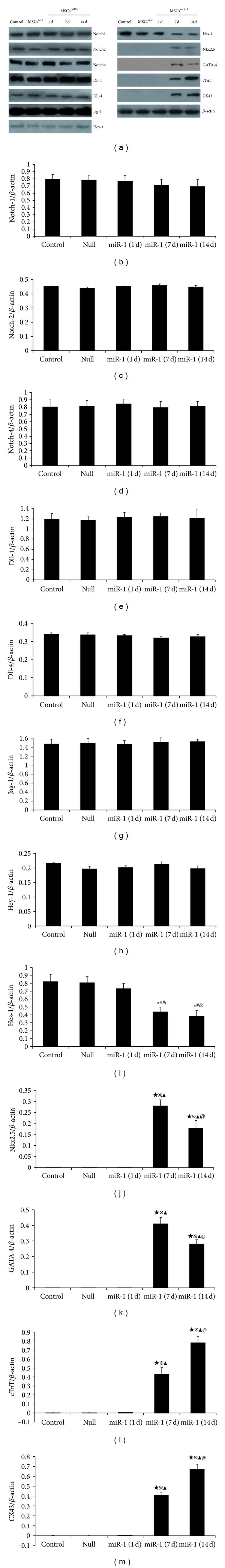
Western blot was performed for Notch signaling and cardiomyocyte-specific markers in MSCs, MSCs^null^, and MSCs^miR-1^ (1 d, 7 d, and 14 d). (a) Expression of Notch-1, Notch-2, Notch-4, Dll-1, Dll-4, Jag-1, Hes-1, and Hey-1 were detected on MSCs. Semiquantitative data showed that the ratio of optical density for Notch-1, Notch-2, Notch-4, Dll-1, Dll-4, Jag-1, and Hey-1 did not alter in MSCs^miR-1^ on days 1, 7, and 14 (b)–(h). The expression of Hes-1 (i) in MSCs^miR-1^ was decreased by days 7 and 14. In MSCs^miR-1^, the expression of Nkx2.5 (j) and GATA-4 (k) were detected on day 7 and decreased by day 14. cTnT (l) and CX43 (m) expression were detected on day 7 and significantly increased by day 14 (control = MSCs; null = MSCs^null^ = MSCs infected with mock lentiviral vectors without miR-1; miR-1= MSCs^miR-1^ = MSCs infected with miR-1 recombinant lentiviral vectors; compared to MSCs, *P < 0.05, ^★^ *P* < 0.01; compared to MSCs^null^, ^#^ *P* < 0.05, ^*※*^ *P* < 0.01; compared to MSCs^miR-1^ (1 d), ^&^ *P* < 0.05, ^▲^ *P* < 0.01; compared to MSCs^miR-1^ (7 d), ^@^ *P* < 0.05).

MSCs revealed expression of Notch-1, Notch-2, Notch-4, Dll-1, Dll-4, Jag-1, Hes-1, and Hey-1 by Western blot (Figure 2(a)). We investigated the change in Notch signaling in MSCs after transfection with miR-1. Semiquantitative data showed that there was no significant difference in expression of Notch-1, Notch-2, Notch-4, Dll-1, Dll-4, Jag-1, or Hey-1 in MSCs^miR-1^ compared to MSCs or MSCs^null^ by days 1, 7, and 14 (all *P* > 0.05) (Figures 2(b)–2(h)). The expression of Notch signaling molecules and cardiomyocyte markers in MSCs^null^ were similar to MSCs at each time point (all *P* > 0.05). This indicated that MSCs only infected with mock lentiviral vectors would not influence the signaling pathway and cells differentiation. However, expression of Hes-1 was decreased in MSCs^miR-1^ on days 7 and 14 (both *P* < 0.01 compared to control)  (Figure 2(i)). To determine the effects of miR-1 on MSCs differentiation on days 1, 7, and 14, we examined MSCs^miR-1^ expression of Nkx2.5 and GATA-4, two transcription factors that are early cardiac markers. We also examined the cardiomyocyte-specific markers —cTnT and CX43 in the same way. In MSCs^miR-1^, Nkx2.5 and GATA-4 expression were detected on day 7 (both *P* < 0.01 compare to control) and decreased by day 14 (both *P* < 0.05 compared to MSCs^miR-1^ (7 d)) (Figures 2(j) and 2(k)). Strikingly, cTnT and CX43 were detected on day 7 (both *P* < 0.01 compared to control) and significantly increased by day 14 (both *P* < 0.05 compared to MSCs^miR-1^ (7 d))  (Figures 2(l) and 2(m)).

**Figure 3:**
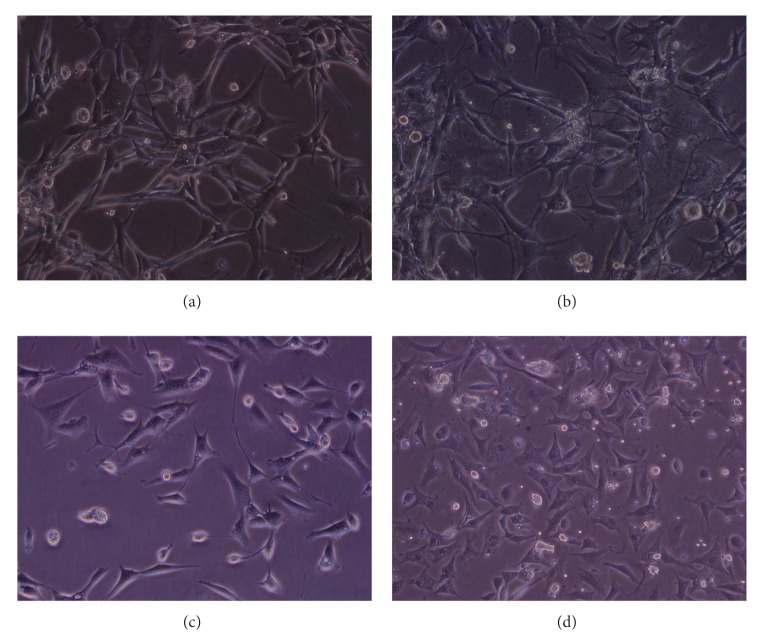
Observation of cell morphology with microscope (Original magnification × 200). (a) MSCs grew as fibroblast-like or long spindle-shaped; (b) 1 day after infected with miR-1, the stem cells had no change in appearance; (c) 7 days after transfected with miR-1, the cells appearance of star or short spindle-shaped; (d)  14 days after modified with miR-1, the cells assume polygonal or short spindle-shaped most look like cardiomyocytes.

The passaged MSCs grew as fibroblast-like or long spindle-shaped (Figure 3(a)). Twenty-four hour after transfected with miR-1, the stem cells had no change in appearance (Figure 3(b)). Seven days after modified with miR-1, the cells assume star or spindle-shaped, with fewer pseudopodia (Figure 3(c)). On day 14, these infected cells appearance of polygonal or short spindle-shaped most looks like cardiomyocytes (Figure 3(d)). Intercalated disc in a little cells was detected by using electron microscope. However, even though overexpression of miR-1 could promote cardiac gene expression in MSCs and most infected cells appearance of cardiomyocyte-like two weeks later, we did not detect any beating cardiac cells during the differentiation process. Maybe MSCs differentiate into cardiomyocytes needed specific conditions besides miR-1. 

### 3.4. Knockdown of Hes-1 Promotes the Expression of Cardiac Makers in MSCs

To study whether downregulation of Hes-1 protein by miR-1 could account for a subset of the effects of miR-1 on cell lineage decisions, we used shRNA constructs directed against distinct regions of Hes-1 to generate Hes-1-shRNA cell line. In our experiment, the Hes-1 mRNA level was about 69% lower in MSCs^Hes-1-shRNA^ than in a control line expressing a scrambled shRNA construct. 

By using Western blot methods, we show that the expression of Hes-1 is significantly decreased in MSCs^Hes-1-shRNA^ on days 7 and 14. Nkx2.5 and GATA-4 expression were induced in MSCs^Hes-1-shRNA^ on day 7, but they were not detected in MSCs. Furthermore, cTnT and CX43 were expressed on day 7 and at even higher levels on day 14 in MSCs^Hes-1-shRNA^ cells. Both cTnT and CX43 were negative for expression in controls. Although the effect of Hes-1 knockdown on expression of Nkx2.5, GATA-4, cTnT, and CX43 was not as robust as the expression in MSCs^miR-1^, the trends were similar. 

## 4. Discussion

MSCs have been used to regenerate cardiac tissues by virtue of their capability to transdifferentiate into cardiomyocytes. Improving the survival and differentiation of transplanted cells in the infarction site is critical for improving the efficiency of stem cell therapies. Notch signaling pathway is involved in many differentiation processes and lineage decisions in fetal and postnatal development. Signaling is initiated via ligand-receptor interactions on neighboring cells. The interaction leads to a series of successive proteolytic cleavages. First, extracellular cleavage of Notch occurs by TACE (TNF*α* (tumour necrosis factor *α*-) converting enzyme). This is followed by transmembrane cleavage by *γ*-secretase, releasing the NICD (Notch intracellular domain), which then translocates to the nucleus and heterodimerizes with the transcriptional regulator RBP-J*κ* (recombinant signal-binding protein 1 for J*κ*), converting it from a transcriptional repressor to an activator. NICD binding to RBP-J*κ* replaces the corepressor transcriptional complex with a coactivator complex, which in turn triggers the transcription of Notch downstream target genes, such as bHLH proteins: Hes and Hey [30, 31]. Notch signaling has been shown to direct cells toward alternate differentiation fates [32]. The contribution of Notch signaling in regulation of cardiac marker gene expression has been supported by various reports demonstrating a Notch-mediated suppression of cardiomyogenesis in *Xenopus*, *Drosophila*, and ESCs [23, 33, 34]. Modulation of Notch-1 signaling in MSCs may improve MSCs function in terms of mobilization or recruitment to the injured heart [9]. It has been shown that Notch signaling influences the cell fate decision between mesodermal and neuroectodermal cell fates during ESCs differentiation and downregulation of Notch-1 signaling could induce cardiogenesis in ESCs [23]. Our study demonstrated that the expression of Notch molecules: Notch-1, Notch-2, Notch-4, Dll-1, Dll-4, Jag-1, Hes-1, and Hey-1 were detected in MSCs. Knockdown of Hes-1 contributes to the induction of early cardiac makers, Nkx2.5 and GATA-4, and cardiomyocyte-specific makers, cTnT and CX43 in MSCs. Basing on this finding, one may speculate that Hes-1 plays a critical role in promoting the expression of cardiac gene in the stem cells.

MiRNAs regulate gene expression and act as important factors in the regulation of stem cell function [35–38]. They are also involved in controlling cell fate, and it is likely that they regulate these decisions by regulating numerous genes and pathways. MiR-1, a muscle-specific miRNA, has been suggested to play a role in cardiogenesis and cardiac gene expression [11, 12]. The study of Kwon et al. had previously shown in *Drosophila* that miR-1 directly targets the Notch ligand delta for repression [12]. In our study, two orthologs of *Drosophila* delta were identified in mouse MSCs—Dll-1 and Dll-4. Overexpression of miR-1 promotes differentiation of MSCs into the cardiac lineage. However, our semiquantitative data showed that the Notch ligand delta did not alter during the MSCs^miR-1^ differentiation process (on days 1, 7, and 14). In the meanwhile, other Notch upstream molecules, Notch-1, -2, -4, and Jag-1, did not change in the MSCs^miR-1^ at the same time points. These results indicated that miR-1 does not influence the Notch upstream molecules of mouse MSCs. Interestingly, expression of Hes-1 was decreased in MSCs^miR-1^ on days 7 and 14. And specific knockdown of Hes-1 can lead to similar cell fate trends as miR-1 overexpression in MSCs. These results were similar to the recent reports which indicated that Notch-1 inhibition promotes cardiac differentiation [23, 24] and the experiment of Ivey et al. that miR-1 regulates ESCs differentiation to cardiac lineage due to miR-1 inhibiting Dll-1 [25]. Yet our study demonstrated that it is the Notch downstream target molecule, Hes-1, but not Notch-1 or Dll-1, which directly contributes to the induction of cardiogenesis in the mouse MSCs. MiR-1 promotes the differentiation of MSCs into the cardiac lineage due to negatively regulation of Hes-1. This effect might depend on miR-1 directly repressing Hes-1. However, as previously described, Notch signaling is initiated via ligand-Notch receptor interactions on neighboring cells. The interaction leads to a series of successive proteolytic cleavages, which in turn triggers the transcription of Notch downstream target genes. Since miR-1 does not alter Notch ligands and receptors during the differentiation process of MSCs^miR-1^, one may speculate that the Notch signaling system may crosstalk with others signaling pathways such as Wnt, BMP, and TNF-*β*, which have been well characterized as enhancing the expression of cardiac markers in stem cells [3, 5, 39, 40]. Maybe miR-1-mediated induction of cardiogenesis in MSCs due to regulation of other signaling pathways, thereby negatively influence Hes-1.

Even though overexpression of miR-1 could promote cardiac gene expression in MSCs, we did not detect any beating cardiac cells during the differentiation process of cultured MSCs modified with miR-1. One possibility is that MSCs differentiate into cardiomyocytes only under specific conditions, such as myocardial microenvironment. Maybe miR-1 only play a partial role in or accelerate the differentiation process. 

Growing evidence demonstrate that bone marrow-derived MSCs have been proposed as a novel therapeutic approach for improvement of infracted heart function through regeneration of myocardium. However, low survival and differentiation rate of transplanted cells in ischemic myocardium influence the outcome of stem cell transplantation for treatment of the ischemic disease. Cell sheet grafts with genetically engineered properties to promote the differentiating into the cardiac lineage may offer a potential approach to repair dead or injured myocardium. Whether the survival and differentiation of transplanted miR-1 modified cells to the infarction site would improve the efficiency of stem cell therapies needs further investigation.

## 5. Conclusion

In conclusion, our results indicate that the muscle-specific miRNA, miR-1, promotes the differentiation of MSCs into the cardiac lineage by repression of Hes-1. MiRNAs may offer a means to direct the differentiation of MSCs into desired fates. MSCs genetically modified with miR-1 could significantly advance the efficacy of stem cell differentiation.
