# Corrigendum to “miR-1-Mediated Induction of Cardiogenesis in Mesenchymal Stem Cells via Downregulation of Hes-1”

**DOI:** 10.1155/2016/8510747

**Published:** 2016-09-14

**Authors:** Feng Huang, Liang Tang, Zhen-fei Fang, Xin-qun Hu, Jia-yi Pan, Sheng-hua Zhou

**Affiliations:** Department of Cardiology, The Second Xiangya Hospital of Central South University, Hunan, Changsha 410011, China

In Figure 2(a) of the published article entitled “miR-1-Mediated Induction of Cardiogenesis in Mesenchymal Stem Cells via Downregulation of Hes-1,” [1] the authors admit to being careless in altering nonspecific belts, stains, or scratches in the Nkx2.5, cTnT, and CX43 panels. The original figures are presented here.

## Figures and Tables

**Figure 2:**
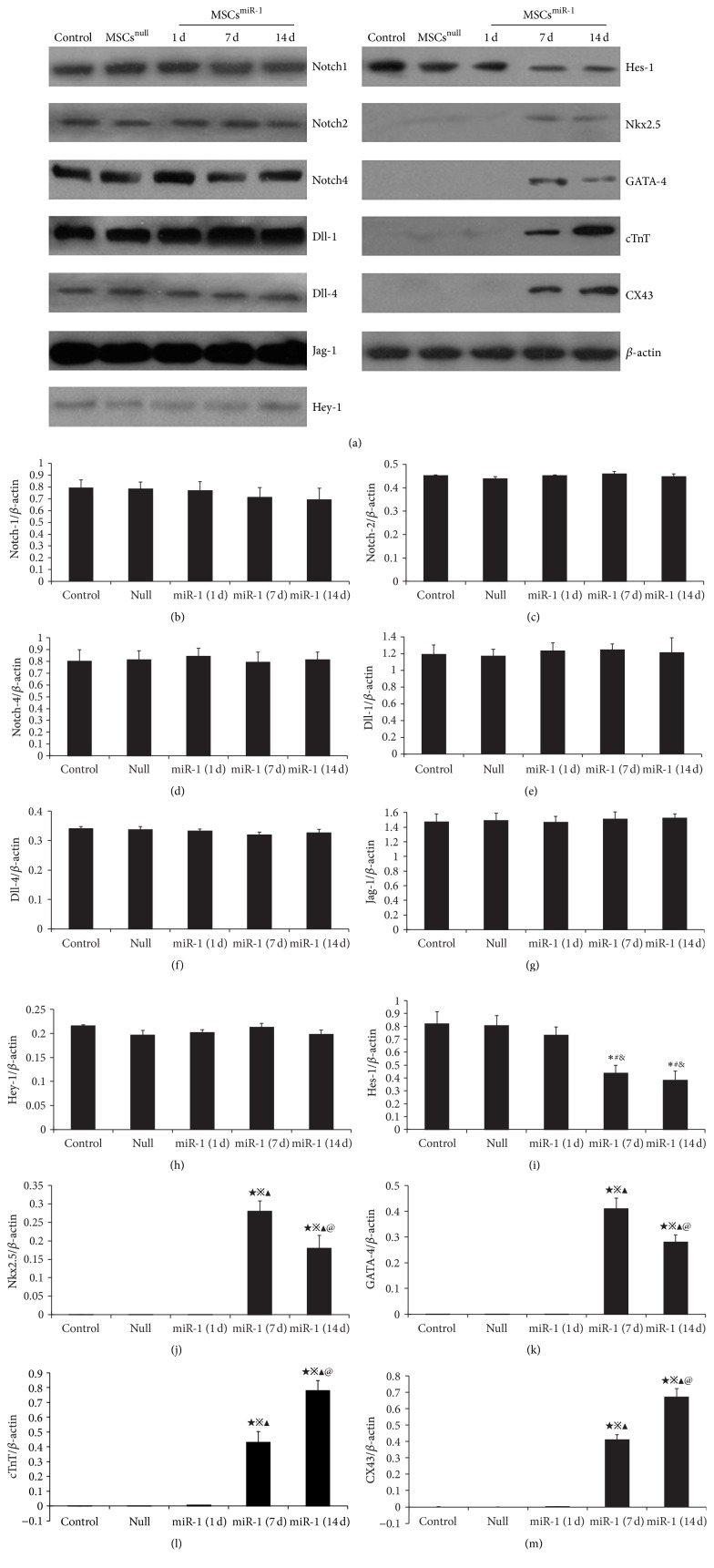
Western blot was performed for Notch signaling and cardiomyocyte-specific markers in MSCs, MSCs^null^, and MSCs^miR-1^ (1 d, 7 d, and 14 d). (a) Expression of Notch-1, Notch-2, Notch-4, Dll-1, Dll-4, Jag-1, Hes-1, and Hey-1 were detected on MSCs. Semiquantitative data showed that the ratio of optical density for Notch-1, Notch-2, Notch-4, Dll-1, Dll-4, Jag-1, and Hey-1 did not alter in MSCs^miR-1^ on days 1, 7, and 14 (b)–(h). The expression of Hes-1 (i) in MSCs^miR-1^ was decreased by days 7 and 14. In MSCs^miR-1^, the expression of Nkx2.5 (j) and GATA-4 (k) were detected on day 7 and decreased by day 14. cTnT (l) and CX43 (m) expression were detected on day 7 and significantly increased by day 14 (control = MSCs; null = MSCs^null^ = MSCs infected with mock lentiviral vectors without miR-1; miR-1 = MSCs^miR-1^ = MSCs infected with miR-1 recombinant lentiviral vectors; compared to MSCs, ^*∗*^ *P* < 0.05, ^★^ *P* < 0.01; compared to MSCs^null^, ^#^ *P* < 0.05, ^*※*^ *P* < 0.01; compared to MSCs^miR-1^ (1 d), ^&^ *P* < 0.05, ^▲^ *P* < 0.01; compared to MSCs^miR-1^ (7 d), ^@^ *P* < 0.05).
